# Certification of NIST SRM 1962: 3 μm Diameter Polystyrene Spheres

**DOI:** 10.6028/jres.097.007

**Published:** 1992

**Authors:** Arie W. Hartman, Theodore D. Doiron, Joseph Fu

**Affiliations:** National Institute of Standards and Technology, Gaithersburg, MD 20899

**Keywords:** electron microscopy, microspheres, optical microscopy, particle sizing, polystyrene, standard reference materials

## Abstract

This report describes the certification of SRM 1962, a NIST Standard Reference Material for particle diameter. It consists of an aqueous suspension of monosize 3 (μm polystyrene spheres. Two calibration techniques were used: optical microscopy and electron microscopy. The first one gave a mean diameter of $\bar{D}=2.977\pm 0.011$ μm and a standard deviation of the size distribution *σ_D_* = 0.020 μm, based on measurement of 4600 spheres. The second technique gave $\bar{D}=2.990\pm 0.009$ μm, based on measurement of 120 spheres. The reported value covering the two results is $\bar{D}=2.983$ μm with a maximum uncertainly of 0.016 μm, with *σ_D_*=0.020 μm.

## 1. Introduction and Summary

This report contains the procedures, measurement results, and error analysis for the certification of SRM 1962, a Standard Reference Material (SRM) for particle diameter. The SRM consists of a 0.5% aqueous suspension of monosize polystyrene microspheres with a mean diameter of nominal 3 μm.

The calibration was carried out by two independent methods: specialized forms of optical and electron microscopy. The two methods are described in Sec. 2, the measurement results are shown in Sec. 3, and the error analysis is given in Sec. 4.

The results of the calibration are as follows:

Optical microscopy:
Mean diameter: 
$\bar{D}=2.977\pm 0.011$ μmDiameter distribution:Gaussian from 10 to 90%Standard deviation *σ*_D_ = 0.020 μmNumber of outliers (defined here as 
$\left|D-\bar{D}\right|>4$
*σ*_D_:< 0.5% for oversize nil for undersize

Seven samples totaling over 4600 spheres were measured.

Electron microscopy:

Mean diameter: $\bar{D}=2.989\pm 0.009$ μm

Reported mean diameter value: $\bar{D}=2.982\pm 0.016$ μm.

## 2. Methods

The two methods used in the calibration of SRM 1962 are described in this section.

### 2.1 Optical Microscopy (CDF)

**Fig. 1:**
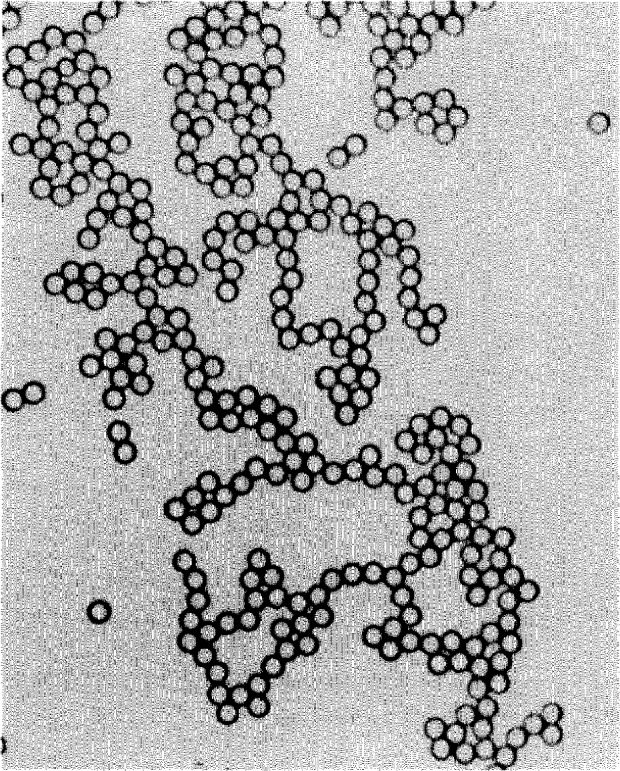
Strands and clusters of 3 μm spheres.

**Fig. 2:**
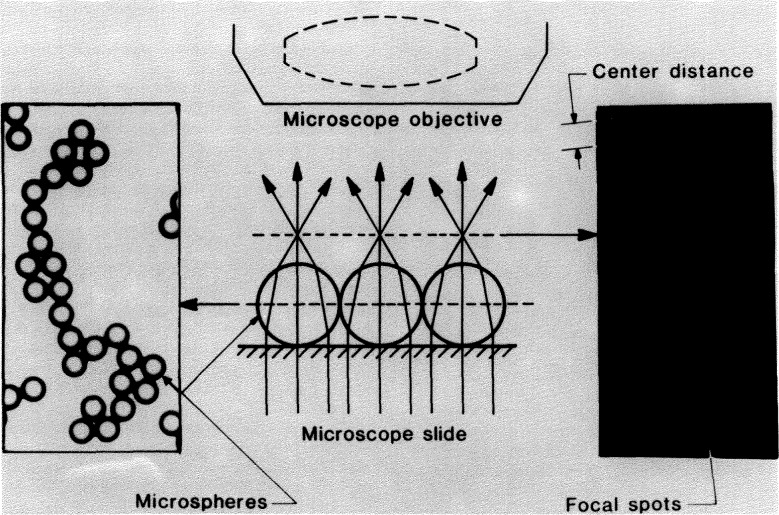
The CDF microsphere sizing scheme.

A drop of microsphere suspension is placed on a microscope slide, and allowed to flow out and dry. During drying the drop breaks up into numerous smaller droplets that dry individually. The spheres that these droplets contain are pulled together by surface tension forces, resulting in strands and small clusters of contacting spheres (Fig. 1). The contacting spheres are illuminated in the microscope by near-parallel light (condenser stopped down), and a number of small and circular “focal spots” form in the common back focal plane, as shown in Fig. 2. When a photomicrograph is taken of this back-focal plane, each recorded spot marks a sphere center. The distances *C* between adjacent spots represent the sum of two sphere radii. If the sphere diameters *D* are distributed normally (Gaussian), the *C*-values will also be distributed normally. The mean value $\bar{C}$ then equals $\bar{D}$ and the standard deviation *σ_C_* of the *C*-distribution equals $\sigma_{D}\sqrt{2}$. In this way $\bar{D}$ and *σ_D_* are found. This technique is called Center Distance Finding, or CDF [1].

The photographed focal spots are small (about 0.5–0.6 μm in the object plane), uniform and circular, permitting center distances *C* to be measured with high precision: a few hundredths of a micrometer in the object plane. It thus allows a measurement of the diameter distribution, which would be difficult to do from measurements of the sphere images themselves.

To make the CDF measurements a number of microsphere slides are prepared and photographed. A large number of photographs are measured under computer control (see Appendix A), The film scale (image magnification) is measured, as outlined in Appendix B. The Image distortion, which for high-quality optics is a function of off-axis distance only. Is measured also (see Appendix B). The computer then applies a radial correction to each measured focal spot position. The corrected center distances *C* are determined, which leads to $\bar{D}$ and *σ_D_.*

### 2.2 Electron Microscopy (MEM)

With this method, called Metrology Electron Microscopy or MEM, the focused beam of a scanning electron microscope (SEM) is held stationary while a single-axis scanning stage with interferometric position readout moves the specimen such that the focused electron beam traverses the distance between leading and trailing edge of the sample to be measured. In our case the sample consists of a straight row of equal-size microspheres.

An interferometer system measures stage travel versus time during a constant-speed scan, and the secondary electron detection system measures the electron output varying with time, all under computer control. The two data streams are combined resulting in a value for the length of the measured microsphere row, from which an average sphere diameter is found. The operation resembles that of an optical measuring microscope, where a set of crosshairs defines a stationary reference point in the field of view and a micrometer screw measures stage travel. See also Refs. [2] and [3].

## 3. Measurements

In this section details are given of the specimen preparation, data collection and reduction, and the measurement results. Section 3.1 covers optical microscopy, Sec. 3.2 treats electron microscopy.

### 3.1 Optical Microscopy (CDF)

Four samples were taken from one vial of SRM 1962 microsphere suspension, and one sample from each of three other vials. The vial contents were homogenized by rolling and shaking for 2 min, prior to dispensing a drop of suspension for analysis.

The microscope used was an Olympus Model BH-2^1^ with a 100×/0.90NA objective, producing images with 1000 × magnification on 4 × 5 in Polaroid sheet film.

**Fig. 3:**
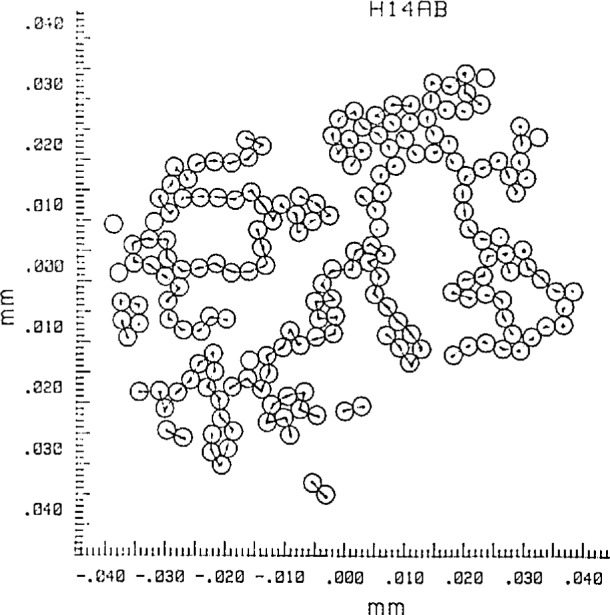
Measurement path for a sphere grouping.

Focal-spot patterns from the contacting microsphere structures were photographed on Type 57 positive film. This high-speed material (3000 ASA) has adequate dimensional stability [1] and low granularity, permitting its use for this SRM calibration. Seventy-six photographs were measured, containing over 4600 measured focal spots. The measurement path through each microsphere grouping was selected such that each sphere was measured only once. The groupings of contacting spheres were examined first for overdeterminedness, to indicate where small air gaps between apparently contacting spheres could have formed during the drying process. Such gaps have minimum widths ranging from zero to typically one *σ*_D_. Air-gap formation can occur in contacting microsphere groupings, such as hexagonal arrays where six neighboring spheres surround a center sphere while the spheres have slightly different diameters [4,5]. Such overdetermined sphere groupings were avoided in the measurement phase. An example of a selected measurement path is given in Fig. 3.

The measured photographs had a print magnification of nominal 1000 ×. The measured focal spots had 0.5–0.6 mm diameters, their 3 mm center spacings were measured with 0.01 mm resolution. The microscope image calibration for magnification and image distortion is detailed in sec. 4.1.1 and in Appendix B.

**Fig. 4:**
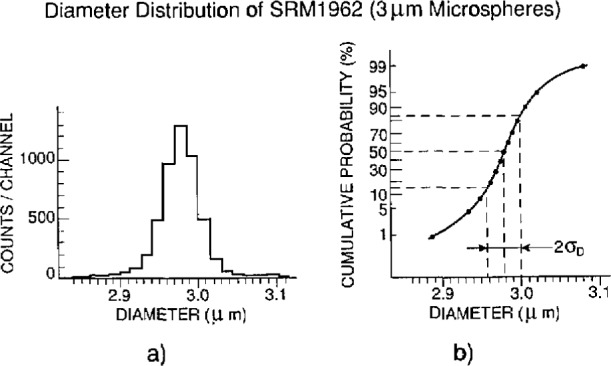
SRM 1962: a) diameter distribution; b) cumulative distribution.

Measurement results are given in Table 1 and in Fig. 4. The data were originally plotted with center distances as the horizontal axis. This was then converted into a diameter scale by compressing the horizontal scale by $\sqrt{2}$ to reflect the fact that for normal distributions $\sigma_{D}=\sigma_{C}\sqrt{2}$, and by centering the D-scale such that the mean diameter $\bar{D}$ coincides with the mean center distance $\bar{C}$. The resulting “diameter distribution” of Fig. 4a already implies that this distribution is considered a normal one. The information extracted from Fig. 4 is: a) the median diameter (which corresponds with the average diameter $\bar{D}$ if the distribution is normal), b) the diameter range over which it actually is normal, and c) the value for the standard deviation *σ_D_* associated with that diameter range.

### 3.2 Electron Microscopy (MEM)

The contents of an SRM 1962 vial were homogenized by rolling and shaking for 2 min. Then a drop was taken from the vial, diluted in 50 ml of 18 MΩ cm deionized water, and washed three times to reduce the amount of dissolved material remaining (biocide). Each washing cycle involved low-power ultrasonication, settling and decanting four-fifths of the clear liquid. A small drop of the final suspension was placed on three microscope slides and air dried, causing formation of single-layer arrays with hexagonal ordering. The slides were then coated with about 30 nm of amorphous carbon to minimize charging in the electron beam. With this technique the formation of sphere arrays was sought, as opposed to the case of CDF (see Sec. 4.2.1).

The electron microscope used for the microsphere diameter measurements is a Vacuum Generators VG HB-50A scanning electron microscope. It has in the secondary electron imaging mode an edge resolution of 0.03 μm at 30 keV and a 25 mm working distance. The interferometer is a single-pass polarization Michelson type, mounted in the SEM vacuum on the fixed and moving parts of a piezo-electric scanning stage. The two reflectors are corner cube prisms to accommodate the relatively long distance (some 80 cm) from the stage inside the SEM column to the interferometer readout system outside. The scanning stage is placed on top of the X-Y stage in the SEM. The X-Y stage is used for searching.

**Fig. 5:**
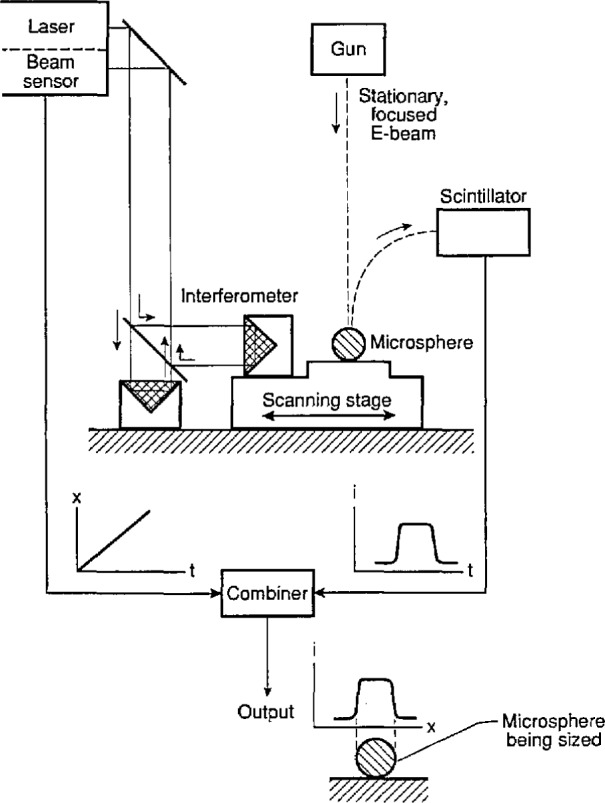
Diagram of the Metrology Electron Microscope (MEM).

The interferometer readout is a Hewlett-Packard Model 5526A, utilizing a two-frequency stabilized He-Ne laser and a heterodyne scheme for measuring optical path differences. The two reflectors are mounted in the SEM vacuum on the fixed and moving parts of the piezo-electric one-axis scanning stage. The reflectors are corner cube prisms, to accommodate any misalignment over the relatively long distance (some 80 cm) from the stage inside the SEM column to the interferometer readout system outside. A block diagram of the MEM system is given in Fig. 5.

**Fig. 6:**
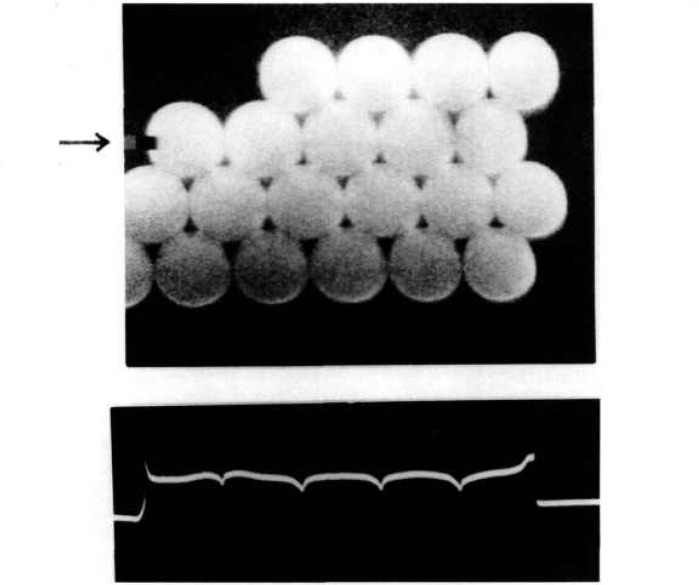
Measuring microspheres with MEM.

Forty microspheres were measured on each of three slides, selecting array rows that were essentially parallel to the scanning stage axis. The rows were at least 12 spheres long allowing measurement of the spacing between two contact planes separated by 10 spheres, from which an average sphere diameter was calculated. Visibly obvious outliers were excluded from the measurements. After each computer-controlled scan along a microsphere row the microscope was reverted to scan mode (SEM mode) and the next sphere row positioned manually for a line scan (spot mode). The scans were 60 μm long, with the secondary electron intensity profile being sampled at 2000 equispaced points. Each minimum in SEM output current signals the passing of a contact plane between two touching spheres (see Fig. 6). Each minimum fell within one data point spacing. Measurement results are given in Table 2.

## 4. Error Analysis

In this section sources of uncertainty (called “errors” for short) are identified and evaluated for the two microsphere size measurement techniques. They are expressed as “3 *σ*” or “maximum” errors as indicated, the individual independent contributions are summed in quadrature, and the total systematic and random errors are added linearly to form “the uncertainty” of the measurement process (see also Tables 3 and 4).

### 4.1 Errors in Optical Microscopy

The errors in measuring the average diameter can be arranged in three groups: errors associated with finding the image magnification of the measured photographs, errors associated with measuring photographed focal spot spacings (center distances between contacting spheres), and errors associated with the diameter distribution. To find estimates for the first two errors, five repeat photographs were taken. Averaging of the repeat data was done to find the magnification at lower uncertainty, while comparison between the photographs was used to find scatter in measured focal spot spacings from which uncertainties in the magnification and in a single measurement of center distance can be derived. The three groups of errors are discussed separately.

#### 4.1.1 Errors Associated with Image Magnification

The print magnification was found by photographing an interferometrically calibrated chrome-on-glass stage micrometer (NIST No. 5525). The line center spacings were measured on a SGIP Universal Measuring Machine, Model MU-214B. The measured and averaged lengths are corrected for image distortion which had been measured separately (Appendix B). The result was an image magnification value valid over the whole field of view, this value is equal to the on-axis value prior to image distortion removal. A number of error sources affected the result, as detailed below.

- The object micrometer.

A length 2.10–2.20 mm of the micrometer mentioned before was used. The length of segment 0–2.11 mm is 2100.58 μm with a maximum error of 0.17 μm that of segment 0–2.20 mm was 2199.51 ± 0.11 μm, giving for 2.11–2.20 mm a length 88.93 ±0.20 μm. This corresponds to ±0.227% or 0.0068 μm for a 3 μm length in the object plane, a systematic error.

- The SGIP film measuring machine.

The scale error amounted to approximately 1.3 μm maximum per setting, or about 2 μm for a difference between two settings. For the measured film distances (89.271 mm mean value) this amounts to about 0.002% or 0.0001 μm in the object plane, a systematic error.

- Film emulsion shifts, image magnification scatter, and film readout errors.

Polaroid Type 57 positive film exhibits local random emulsion shifts, like most photographic emulsions. These lateral shifts are caused by non-uniform film processing and drying.

Magnification scatter is caused by slight changes in film position in the cassette (measured along the optical axis) when exposed film is replaced by a new film sheet.

Film readout errors reflect the precision with which one can visually pinpoint the center position of the scale division lines of the photographed object micrometer.

The combined contribution by these three error sources was found as follows. The utilized 0.09 mm section of the calibrated object micrometer was photographed five times at 1000 ×, giving image lengths (in mm): 89.807, 89.924, 89.863, 89.903, and 89.607. The mean was 89.837, with a 3 *σ* scatter of 0.142 mm or 0.159%. This total scatter contributes a 0.0048 μm error to a 3 μm center distance measurement. After correction for image distortion, see d) below, the mean becomes 89.271 mm, giving an on-axis image magnification *M*_o_ = 89.271 mm/88.93 μm = 1004×.

- Image distortion.

The microscope used exhibits radial image distortion: each off-axis image point is shifted radially by a small amount from its intended position. In our case each end point of the 89 mm measured length was shifted outwards by 0.30 mm typically, with an estimated maximum uncertainty of 0.030 mm (Appendix B). This amounts to a combined 0.060 mm uncertainty in the measured length (the two error contributions are correlated), or 0.067%, corresponding to 0.0020 μm in the object plane.

The error contributions a) through d) combine to a total systematic error of 0.0089 μm (see Table 3).

#### 4.1.2 Errors Associated With the Determination of Microsphere Center Distances

The accuracy of center distance measurements is affected by various uncertainties: those associated with (a) pin-pointing the positions of focal spot centers in the film, (b) with the correction of measured focal spot positions due to image distortion, (c) with the fluctuations in print magnification when new film is inserted in the cassette, (d) with possible distortion of the spheres at the contact areas, and (e) with the possibility that the individual spheres might be slightly deformed (showing a non-circular cross section when measured perpendicular to the line of sight).

- The combined effect of film readout (pinpointing sphere centers) and emulsion shifts was found by taking three repeat exposures of a hexagonal array of the 3 μm spheres, and measuring each time the same 17 distances between adjacent sphere centers in a selected microsphere row, under computer control as described in Appendix A. All 17 sets of three center distance readings each were scaled down to the same average value (nominally 3.0 mm, as a result of 1000 × magnification of the 3 μm sphere objects). The 51 values were then pooled, resulting in a 3 *σ* scatter of 17.3 μm which amounts to 0.017 μm in a 3 μm object distance, a random error. As can be seen, this procedure reduced the effects of magnification scatter and avoided the effects of off-axis magnification changes due to image distortion and of unequal-size spheres.
  When pinpointing the center positions of the focal spot recordings in the film the utilized coordinate measuring machine with TV-microscope probe exhibited a reproducibility of better than 0.5 μm at 1 *σ* (see Appendix A). This translates to a maximum error of 2 μm in film distances between two focal spots, or 0.002 μm in distances between microsphere centers. This random error is included in the above error discussion, and does not noticeably increase the 0.017 μm random error calculated.
- The effect of image distortion in our case (see Appendix B) is maximum for a sphere pair at the edge of the measured field of view. At 40 mm off-axis distance the maximum error in the measured image distortion is about ±20 μm, at 37 mm it is ± 16 μ. Assuming as a worst case that these errors are uncorrelated, the resultant maximum error for 3 mm center distances near the edge of the 80 mm field of view will be ± 25 μm or 0.025 μm in the object plane, a random error. For center distances closer to the optical axis this error will be considerably less, and for those on the axis the error will be zero.
- Magnification scatter, occurring when replacing sheet film in the cassette, was measured as 0.27% at 3 *σ* for the central area of a single exposure (see Appendix B). This value is considerably larger than can be expected from the data in c) of Sec. 4.1.1, A reason is that c) relates to measurements near the edges of the film sheet (the imaged object micrometer segment filling the field of view), where it is clamped by the cassette mechanism and consequently flexes much less. The corresponding maximum error for a 3 μm center distance measurement is 0.008 μm, an essentially random error. It has been applied to all areas in the film, as a worst case.
- One can adapt the model that two polystyrene spheres approaching each other during the drying process will finally be in intimate contact over a circular area, the extent of which is controlled by a balance between van der Waals attraction and elastic deformation. This model has been analyzed by Derjaguin et al.; they have derived an expression for the resultant sphere flattening [6]. For the present case the two-sided flattening would amount to a shortening Δ*C* of the measured center distance *C* given by
  $$\Delta C=\frac{1}{8}{\left[\frac{6{\left(1-\eta^{2}\right)}^{2}DA^{2}}{\in^{4}E^{2}}\right]}^{1/3}$$,
  in which
  *η*: = Poisson constant, 0.3 for polystyrene
  *D*: = sphere diameter, 3 × 10^−4^ cm
  *A*: =Hamaker constant, 1 × 10^−12^ erg for polystyrene
  *E*: = Young’s Modulus, 3 × 10^10^ dyne/cm^2^ for polystyrene
  *ϵ*: = distance of closest approach, 3 × 10^−8^ cm.
  This gives Δ*C* = 1.3 nm = 0.0013 μm, lowering the measured diameter. If the selected values for *A* and *E* are each uncertain by a conceivable factor 2, then Δ*C* could change by a factor whose maximum value is $3\sqrt{16}=2.5$. The Δ*C* estimate then ranges from 0.0005 to 0.003 μm.
  Although this model for sphere flattening on contact is not the only one [7] available, experimental data (comparison with other calibration techniques for various monosize microsphere Standard Reference Materials) support the Derjaguin model. Therefore the measured diameter values in Table 1 are corrected afterwards by a somewhat arbitrary increase of 0.002 μm, and a systematic error 0.002 μm is entered in the Error Analysis.
- If a microsphere is elongated perpendicular to the line of sight, its focal spot will be elongated by the same amount [1]. The photographed focal spots are almost all very uniform and circular, with a diameter of 0.4–0.5 mm in the film plane corrersponding to 0.5 μm in the object plane. A non-circularity of 0.03 mm is visually detectable, and any residual non-sphericity will then not exceed 0.030 μm—a random error.
  The random contributions combine to a maximum random error of 0.043 μm.

#### 4.1.3 Errors Associated With the Microsphere Diameter Distribution

Figure 4b shows that the diameter distribution is not quite normal. Of the measured population 98% covers the size range 2.89 to 3.08 μm. The maximum error contribution to a single center distance measurement can be set at ±0.095 μm–a random error.

#### 4.1.4 Combining the Various Error Contributions for the $\bar{D}$ Measurement

From Table 3 the total random error amounts to $0.104/\sqrt{4600}=0.0015$ μm, the total systematic error is 0.0091 μm, therefore the total error in $\bar{D}$ is 0.011 μm, giving $\bar{D}=2.977\pm 0.011$ μm.

#### 4.1.5 Finding the Standard Deviation *σ_D_* of the Size Distribution

Figure 4 shows that the diameter distribution is normal from 10 to 90% (3700 spheres), and the calculated value of *σ_D_* for that population is 0.021 μm with a statistical uncertainty in *σ_D_* of ± 14% at 3 *σ*, or 0.003 μm.

Subtracting in quadrature the 1 *σ* random uncertainty in a single measurement of center distance (equal to 0.043/3 μm or 0.014 μm, see Table 3) would lower *σ_D_* to 0.017 μm, but the uncertainty in this value will increase correspondingly.

The reported value for *σ_D_* has been set at 0.020 μm.

### 4.2 Errors in Measuring Microsphere Average Diameter by Electron Microscopy

These error contributions can be divided into four groups: errors associated with the microsphere sensing process, errors associated with the measurement, of scanning stage travel, errors associated with the kind of grouping the measured microspheres are in (single-layer microsphere arrays with hexagonal ordering), and errors associated with the microsphere diameter distribution width.

#### 4.2.1 Errors Associated With Microsphere Sensing

**Fig. 7:**
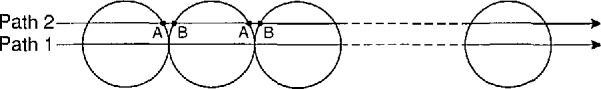
Row length errors (see text).

- Imperfect scan conditions.
  Referring to Fig. 7, path 1 represents a possible scan path, while path 2 produces deeper minima of SEM output at the contact plane position midway of the line segments AB. When measuring contact plane spacings scan 2 gives no additional errors over scan 1.
  When the scan path makes a small angle *α* (radians) with the center line, a cosine error occurs and the measured spacing is too large by a fraction 1/2 *α*^2^. A 10-sphere scan beginning at 1/2 *R* above the center line and ending at 1/2 *R* below it (a worst case), has *α* = 0.05 and will have an error in the calculated scan length of 0.125% or 0.0375 μm. For 12 such scans, covering all 120 measured spheres, the random error in the calculated average diameter $\bar{D}$ is then $\left(0.0375\sqrt{12}\right)/120$ μm, or 0.0011 μm.
  If as a worst case two unequal-size contacting spheres have diameters 2% smaller than $\bar{D}$ and 2% larger than $\bar{D}$ respectively, the midpoint of the segment AB in Fig. 7 shifts a calculated $0.0014\bar{D}$ or 0.0042 μm. If this happens at both ends of the measured contact plane spacing containing *N* spheres, the maximum error in the calculated value for $\bar{D}$ is 0.0028 $\bar{D}/N$. In the present case all 12 measured rows contained 10 spheres each. When all data are pooled the resulting random maximum error in $\bar{D}$ will be 0.0028 $\bar{D}/\left(10\sqrt{12}\right)=0.0002$ μm.
- E-beam exposure.
  If E-beam exposure causes the polystyrene spheres to swell or shrink uniformly, the spheres could conceivably move with respect to each other and with respect to the substrate. A uniform swelling causes in principle increased contact area (sphere flattening) without changing sphere center distances, giving no measurement errors. A uniform shrinking causes in principle no errors if each sphere remains anchored to the substrate, while errors will occur if a sphere can move laterally (either by rolling over the substrate and sliding against its neighbor, or by sliding over the substrate while being in fixed contact with its neighbor).
  Lateral sphere motions were not observed, and in a few cases development of a tiny “neck” or “bridge” was found between adjacent spheres, indicating that a uniform shrinking might be taking place there. Tentatively an upper limit of 0.005D has been placed on row end point shifts caused by sphere motions, leading to a maximum error of 0.01D in the measurement of a 10-sphere contact plane spacing and a systematic error of $0.01\bar{D}/\left(10\sqrt{12}\right)$ or 0.0009 μm in the calculated $\bar{D}\text{-value}$ covering 120 spheres.
- SEM-resolution and E-beam wander.
  The combined effect is estimated at 0.02 μm when pinpointing an individual contact plane or $0.02\sqrt{2}$ for a 10-sphere contact plane spacing. This gives for 120 spheres a random error in $\bar{D}$, equal to $0.02\sqrt{2}\times \sqrt{12}/120=0.0008$ μm.

#### 4.2.2 Errors Associated With Stage Travel (Row Length) Measurements

- Stage travel sampling.
  Each scan is 60 μm long, and covers 2000 equally spaced data points for a least count of 0.033 μm. This gives a sampling error of 0.033 μm when measuring contact plane spacings. For 10-sphere rows and a total of 12 rows the resultant error in $\bar{D}$ becomes 0.0009 μm.
- Interferometer digitizing.
  The interferometer has a least count of *λ*/40, giving an error in contact plane spacing of *λ*/40 and a resultant error in $\bar{D}$ (using *λ* = 0.6328 μm, 10-sphere rows, and 12 rows total) equal to 0.0005 μm.
  The errors associated with the electronics of SEM, interferometer, and computer, are considered negligible.

#### 4.2.3 Errors Associated With Contacting Microsphere Groupings

- Sphere deformation at the contact areas.
  At the contact areas sphere flattening can occur as discussed in Sec. 4.1.2. It is in effect a change in scale, leading to a correction in the measured $\bar{D}$ set at + 0.002 μm, and a systematic error of 0.002 μm.
- Air gaps between spheres.
  Hexagonal arrays of slightly unequal-size spheres exhibit small air gaps throughout the array; the average gap width for normal size distributions is about 0.46 times the standard deviation of the diameter distribution [2,3]. For this microsphere SRM *σ_D_* = 0.020 μm as found in Sec. 3.1, leading to a diameter correction of −0.46 *σ_D_* = −0.0090 μm with an estimated systematic uncertainty of 0.0020 μm.

#### 4.2.4 Errors Associated With Sample Size

The maximum error in the measurement of average diameter of a microsphere material with *σ_D_* = 0.020 μm based on a sample of 120 spheres, will be equal to $3\sigma_{D}/\sqrt{120}=0.0055$ μm—a random error.

#### 4.2.5 Combining the Various Error Contributions to $\bar{D}$

The total random error is found as a linear sum of the sampling and digitizing errors, plus an rms sum of the other random contributions, for a total of 0.0057 μm. The total systematic error is an rms sum of the individual contributions, amounting to 0.0030 μm. The reported total uncertainty in $\bar{D}$ is then 0.009 μm. The mean diameter itself, after applying the corrections for sphere flattening (+ 0.0020 μm) and air gaps (−0.0090 μm), is 2.990 μm.

## *5.* Diameter Calibration Final Results

Optical Microscopy

Average diameter of SRM 1962 microspheres:

$$\begin{array}{ll} \bar{D}=2.977\pm 0.011\mu m & \left(\text{optical}\;\text{microscopy}\right)\\ \sigma_{D}=0.020\mu m & \left(\text{central}\;\text{peak}\right) \end{array}$$

Electron Microscopy

$$\bar{D}=2.990\pm 0.009\;\mu \;\left(\text{electron}\;\text{microscopy}\right)$$

Because the two methods gave noticeably different $\bar{D}\text{-values}$ and have almost the same total uncertainty, the reported value for the average diameter was set at $\bar{D}=2.983\pm 0.016$ μm.

The quoted uncertainties are maximum values.

## 6. Sample Uniformity

From Table 1 an impression of sample uniformity can be obtained: the within-vial variation in the measured mean diameter is less than ±0.1%, and the between-vial variation amounts to a slightly larger (±0.10%) amount. Therefore, we take as an upper limit for the SRM non-uniformity: ±0.1% for within-vial and between-vial sampling.

## 7. Outliers

As with the sample uniformity, only upper limits could be set to the percent oversize and undersize of the measured 2000 spheres. When outliers are defined as spheres with sizes more than *4σ_D_* different from the average diameters, there are about 1% oversize and about 1% undersize (Fig. 4b). Spheres that were outsize by some 10% or more would be detectable by visual inspection of the photomicrographs. No such spheres were found.

## Figures and Tables

**Fig. 8:**
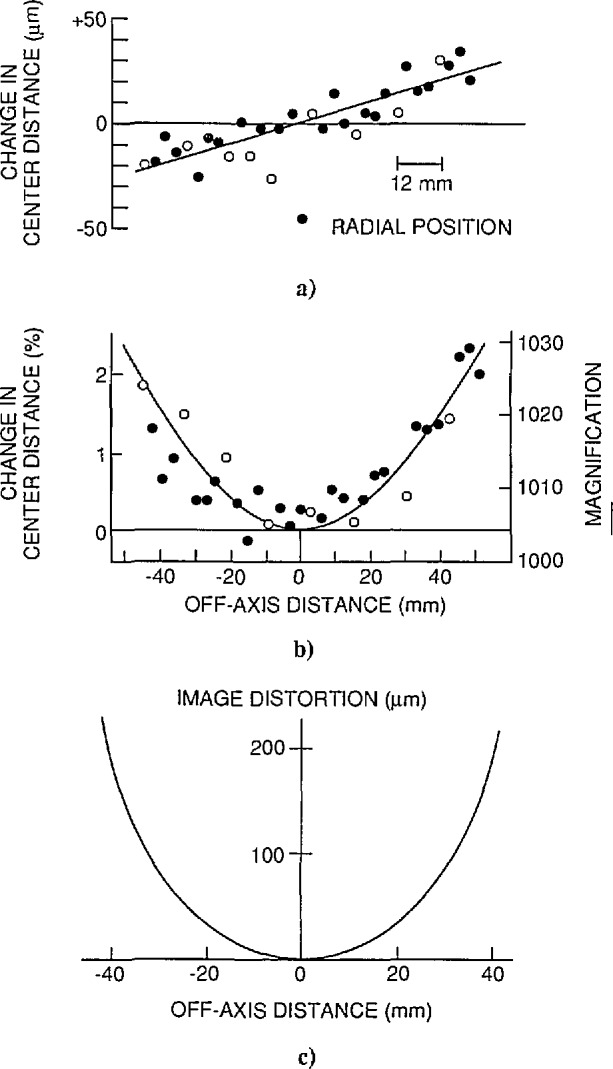
Finding microscope image distortion and magnification.

**Fig. 9:**
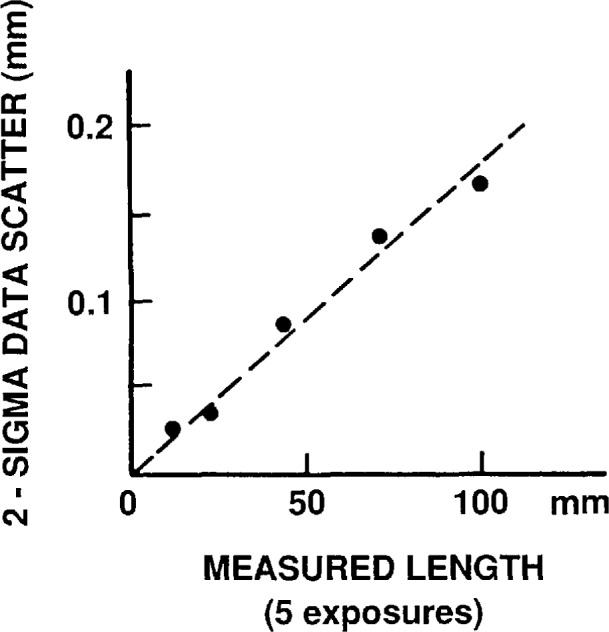
Scatter in image magnification.

**Table 1:** Measurement results with optical microscopy*

Vial#	Sample #	Sphere diameter (μm)	# of measurements	# of photographs
1	1	2.975	1092	15
1	2	2.972	571	9
1	3	2.974	475	11
1	4	2.975	501	17
1	1 to 4	2.974	2639	52
2	5	2.978	720	11
3	6	2.975	214	4
4	7	2.975	1061	9
all	all	2.975	4634	76

a Diameter distribution is approximately normal for 10 to 90%. Standard deviation over this interval: 0.021 μm.

**Table 2:** Measurement results with electron microscopy

Microsphere row #	Average diameter (μm)
1	3.008
2	3.000
3	3.000
4	2.998
5	3.003
6	2.993
7	2.996
8	2.997
9	2.997
10	2.981
11	2.994
12	2.996
Average	2.997

**Table 3:** Error budget for a single 3 μm center distance measurement, using CDP

Category	Error source	Error contribution (μm)		
		Systematic	Random	
On-axis magnification	Stage micrometer calibration	0.0068		
	Film measuring machine calibration	0.0001		
	Film readout, emulsion shifts, and magnification scatter (5 exposures)	0.0053		
	Image distortion uncertainty	0.0020		
		0.0089		
Center distance measurement	Film readout and emulsion shifts		0.017	
	Magnification scatter		0.008	
	Image distortion-worst case		0.025	
	Sphere flattening on contact		0.002	
	Non-sphericity		0.030	
	Subtotals	0.0089	0.043	
Finite sample size (*N* = 4600)	Diameter distribution width		0.095	
	Totals	0.0089	0.104	

a Uncertainty in $\bar{D}/0.0089+0.104/\sqrt{4600}=0.011$ μm. Measured $\bar{D}$ (after correction, see Sec. 4.1.2): 2.977 μm.

**Table 4:** Error budget for an average diameter measurement of 3 μm spheres, using MEM^a^

Category	Error source	Error contribution (μm)	
		Systematic	Random
Microsphere sensing	Imperfect scan conditions: scan direction unequal-size spheres		0.0011 0.0002
	E-beam exposure	0.0009	
	SEM resolution and E-beam wander		0.0008
Row length measurements	Stage travel sampling interferometer least count		0.0009 0.0005
Microsphere grouping	Sphere flattening on contact air gaps between spheres	0.0020 0.0020	
Sample size (*N*=120)	Small sample with non-zero *σ_D_*		0.0055
	Totals	0.0030	0.0057

a Uncertainty in $\bar{D}:0.0030+0.0057=0.009$ μm. Measured $\bar{D}$ (after corrections, see Sec. 4.2.5): 2.990 μm.

## Footnotes

1 Certain commercial equipment, instruments, or materials are identified in this paper to specify adequately the experimental procedure. Such identification does not imply recommendation or endorsement by the National Institute of Standards and Technology, nor does it imply that the materials or equipment identified are necessarily the best available for the purpose.

## 8. Appendix A. Measuring Microsphere Center Distances From Photomicrographs

The relative positions of the photographed microsphere focal spot positions are read out with a coordinate measuring machine (CMM). Its probe is a microscope containing a high-resolution image sensor (vidicon) and circuitry to pinpoint the center of each focal spot, which produces steering signals to the X-Y CMM drives to bring the focal spot center to the boresight axis (center of the microscope’s field of view). The CMM’s X-Y coordinates are then stored.

Next, for each photograph being measured a decision is made as to what measurement path will be selected from one focal spot to the next, in order to make sure that each sphere is measured only once and that each sphere in the grouping was free to assume its contacting position with its neighbors during drying (no mechanically overdetermined sphere arrangement). The path selection is done by an operator using an interactive graphics routine. The CRT display shows all focal spots in their relative positions with circles drawn around each one, to simulate the actual microsphere scene rather than the focal spot representation of it. It also shows the keyed-in measurement path (see Fig. 3). The result is a string of X-Y coordinates in which each increment represents a center distance (CD) between two adjacent (contacting) spheres.

Before the CDs are computed each focal spot position is first corrected for image distortion of the microscope (this distortion can be determined as in Appendix B). Because the distortion is a radial function these position corrections take the form of small radial shifts. Their magnitudes depend on the initial off-axis distances, and are found by means of a stored function or lookup table.

An impression of the repeatability and accuracy of the film measuring system can be obtained from the following information:

1. The CMM is normally used for the calibration of precision grid plates. It has been extensively studied, and its positional accuracy is better than 0.1 μm over the area of interest [8].
2. Each focal spot was centered to the same point of the camera field, and all the spots were about the same size. Therefore any geometrical errors in the camera scan can have only secondary effects on the measurement.
3. A photograph selected at random was run a number of times without being moved. The center distances (3 mm nominally, the print magnification being 1000×) showed a repeatability with standard deviation less than 0.5 μm.
4. One other photograph was measured repeatedly while the microscope focus was varied in both directions. Since the focal spot brightness was not completely isotropic, the focus settings changed the apparent size and shape of the focal spots by small amounts. The resultant changes in focal spot positions were very small and uncorrelated, with an estimated standard deviation of less than 0.5 μm.
5. A few photographs were reexamined after all had been measured in order to check for any process changes during the measurements. No changes larger than the measured repeatability were found.

The CMM used was a five-axis Moore V from Moore Special Tools Company. The TV camera system consisted of a Dage-MTI Inc. high-resolution vidicon camera Model 65, with a Bausch & Lomb lens type Mono Zoom 7 and a vision system from Videometrix VPU, Model 101110-501-14. The scene illumination was a diffuse one, using a fiber-optic ring illuminator from Titan Tool Company.

## 9. Appendix B. Measuring the Print Magnification (Film Scale) and Image Distortion

To set the scale of the photographs a section of a calibrated object micrometer is photographed and measured. To maximize resolution the photographed length is made to span the whole field of view. The measured length is then corrected for the effects of image distortion which causes off-axis image points to be radially shifted from their intended positions. The corrected length now shows the image magnification in the absence of image distortion. In other words: one has found the on-axis image magnification value.

For the determination of image distortion the microscope magnification itself is not needed. In the following is shown how these properties were measured.

- Image distortion.
  Following a scheme outlined in Ref. [1] this goes as follows. A row of microspheres is placed such that it crosses the center of the field of view. Its row of focal spots is photographed. Then the row is shifted in-line by four sphere diameter (4*D*) and its focal spots are recorded again. All distances between adjacent sphere centers are measured in both photographs; it will be seen that some center distances will have decreased while others increased. These changes are so small that an in-line shift of 4*D* rather than *D* was chosen in order to make the center distance changes stand out from measurement noise (see Fig. 8a).
  Assuming a 4*D* shift from left to right, and starting with the far left (first) sphere pair, one finds the accumulated length changes when the center distance length *D* is shifted in-line by 4*D* at the various points in the field of view. A second data set is found by repeating the length change calculations starting at the next left sphere pair. A third data set is obtained from the third left sphere pair, and likewise for the fourth set. The four data sets belong to a common curve, and a best fit of all four sets is obtained as shown in Fig. 8b.
  Figure 8b therefore shows by what percentage a center distance *D* will vary as it is shifted all across the field of view (this is equal to the percent change in magnification for radial objects as a function of off-axis distance). A graphic integration then yields the radial shifts of off-axis image points as a function of their off-axis distance, that is, the image distortion as shown in Fig. 8c.
- Film scale.
  With the image distortion known, the film scale or on-axis magnification *M*_o_ is found next as shown in Fig. 8b. If *M*_o_ is measured a number of times the results will show data scatter, typically 0.3% at 3 *σ.* This is caused by small changes in axial position when fresh sheet film is inserted in the cassette. The corresponding scale changes can be reduced by averaging over a number of repeat exposures, for instance five, of the object micrometer.
  The effect of inserting fresh sheet film in the cassette can be measured by placing a row of microspheres such that it crosses the center of the microscope’s field of view, taking five repeat photomicrographs, and measuring a number of sphere center distances in all photographs. The scatter found in these lengths contains the combined effects of film readout, local emulsion shifts and changes in film scale. As shown in Fig. 9 these data approximate a straight line through the origin; the slope of that line represents the scatter in film scale or magnification (0.09% at 1 *σ* in our case), for areas near the center of the photographs. For this experiment a 10 μm microsphere array was centered in the field of view, and the various lengths in Fig. 9 were realized by summing the lengths of a number of adjacent microsphere rows. The found magnification scatter was indicative of film flexure at the central area of the film frame.
